# Corilagin alleviates acetaminophen-induced hepatotoxicity via enhancing the AMPK/GSK3β-Nrf2 signaling pathway

**DOI:** 10.1186/s12964-018-0314-2

**Published:** 2019-01-10

**Authors:** Hongming Lv, Lihua Hong, Ye Tian, Changjiu Yin, Chao Zhu, Haihua Feng

**Affiliations:** 1 Key Laboratory of Zoonosis, Ministry of Education, College of Veterinary Medicine, Jilin University, Xi’an Road 5333#, Changchun, Jilin 130062 People’s Republic of China; 2 Department of Ophthalmology, The Second Hospital of Jilin University, 218 Ziqiang Street, Changchun, 130041 Jilin Province People’s Republic of China; 3 Endodontic Department of Stomatological Hospital, Jilin University, Changchun, 130021 People’s Republic of China; 4 Women and Children’s Health Hospital of Jilin Province, 1051 Jianzheng Street, Changchun, 130061 Jilin Province People’s Republic of China

**Keywords:** Corilagin, Acetaminophen, Acute liver failure, Oxidative stress, Nrf2, AMPK/GSK3β

## Abstract

**Background:**

Acetaminophen (APAP) overdose-induced acute liver failure (ALF) is mainly resulted from uncontrolled oxidative stress. Nuclear factor-erythroid 2-related factor 2 (Nrf2), a key antioxidant transcription factor, is essential for alleviating APAP-induced hepatotoxicity. Corilagin (Cori) is a natural polyphenol compound that possesses effective antioxidant activity; however, the protective effect of Cori on APAP-induced hepatotoxicity is still unknown. The current study aimed to explore whether Cori could mitigate hepatotoxicity caused by APAP and the underlying molecular mechanisms of action*.*

**Methods:**

Cell counting kit-8 (CCK-8) assays, Western blotting analysis, dual-luciferase reporter assays, a mouse model, CRISPR/Cas9 knockout technology, and hematoxylin-eosin (H & E) staining were employed to explore the mechanisms by which Cori exerts a protective effect on hepatotoxicity in HepG2 cells and in a mouse model.

**Results:**

Our findings suggested that Cori efficiently decreased APAP-triggered the generation of reactive oxygen species (ROS) and cell death in HepG2 cells*.* Additionally, Cori significantly induced the expression of several antioxidant enzymes, and this induced expression was closely linked to the upregulation of Nrf2, inhibition of Keap1 protein expression, and promotion of antioxidant response element (ARE) activity in HepG2 cells. Moreover, Cori clearly induced the phosphorylation of AMP-activated protein kinase (AMPK), glycogen synthase kinase-3β (GSK3β), liver kinase B1 (LKB1) and acetyl-CoA carboxylase (ACC). Furthermore, Cori-mediated GSK3β inactivation, Nrf2 upregulation and cytoprotection were abolished by an AMPK inhibitor (Compound C) in HepG2 cells. Lastly, we found that Cori inhibited APAP-induced hepatotoxicity and mediated the expression of many antioxidant enzymes; these results were reversed in Nrf2 ^−/−^ HepG2 cells. In vivo, Cori significantly protected against APAP-induced ALF by reducing mortality and alanine transaminase (ALT) and aspartate aminotransferase (AST) levels, attenuating histopathological liver changes, inhibiting myeloperoxidase (MPO) and malondialdehyde (MDA) levels, and increasing the superoxide dismutase (SOD) content and GSH-to-GSSG ratio as well as suppressing c-jun N-terminal kinase (JNK) phosphorylation. However, Cori-induced reductions in mortality, AST and ALT levels, and histopathological liver changes induced by APAP were clearly abrogated in Nrf2-deficienct mice.

**Conclusions:**

These findings principally indicated that Cori effectively protects against APAP-induced ALF via the upregulation of the AMPK/GSK3β-Nrf2 signaling pathway.

## Background

Acute liver failure (ALF), which is primarily caused by drug-induced liver injury (DILI), has now become a significant public health issue worldwide [1, 2]. Acetaminophen (N-acetyl-p-aminophenol, APAP), a nonprescription drug classified as an antipyretic and analgesic, remedies mild-to-moderate fever and pain. However, APAP overdose gives rise to serious liver injury [3]. It is reported that the induction of APAP-toxicity is related to N-acetyl-p-benzoquinone imine (NAPQI), a reactive metabolite, generated primarily through cytochrome P450 enzymes, that decreases glutathione (GSH) content and increases reactive oxygen species (ROS) accumulation in the liver [4, 5]. During APAP administration, ROS overproduction activates c-jun-N-terminal kinase (JNK), which belongs to mitogen activated protein kinase (MAPK) pathways and exacerbates liver tissue injury [6]. Thus, developing hepatoprotective agents to enhance physiological antioxidant capability or eliminate ROS generation may be a crucial strategy to attenuate liver damage and diseases.

Increasing evidence reveals that natural products counteract oxidative stress by upregulating the nuclear factor-erythroid 2-related factor 2 (Nrf2) pathways, which are essential for the prevention and treatment of many diseases, including acute liver failure [7, 8]. Under normal circumstances, owing to the function of its natural inhibitor protein Kelch-like ECH-associated protein 1 (Keap1), Nrf2 is mainly preserved in cytoplasm [9]. Once exposed to stimuli and pharmacological inducers, however, Nrf2 is separated from Keap1, translocated to the nucleus and combined with antioxidant response elements (AREs), further resulting in the expression of diverse genes with biological activity as antioxidants and in detoxification, such as NAD(P)H: quinone oxidoreductase (NQO1), dismutase (SOD), glutamate-cysteine ligase catalytic/modifier (GCLC/GCLM) subunit and heme oxygenase-1 (HO-1), which are important in the biosynthesis of GSH [9, 10]. In this process, AMP-activated protein kinase (AMPK) can modulate inactivation of glycogen synthase kinase 3β (GSK3β) to promote nuclear translocation of Nrf2 [11]. AMPK is a heterotrimeric serine/threonine kinase that controls energy metabolism, and its activation is a complex process that involves a series of upstream kinases, such as liver kinase B1 (LKB1), and downstream molecules, such as acetyl-CoA carboxylase (ACC) [12, 13]. Once AMPK is activated, it plays an essential role in ameliorating APAP-induced ALF [14]. Additionally, several previous reports have indicated that GSK3β, as a novel regulator of Nrf2, is also involved in promoting the regeneration of liver in mice after severe hepatotoxicity induced by APAP [11, 15], suggesting cooperation between AMPK/GSK3β signaling and Nrf2.

**Fig. 1:**
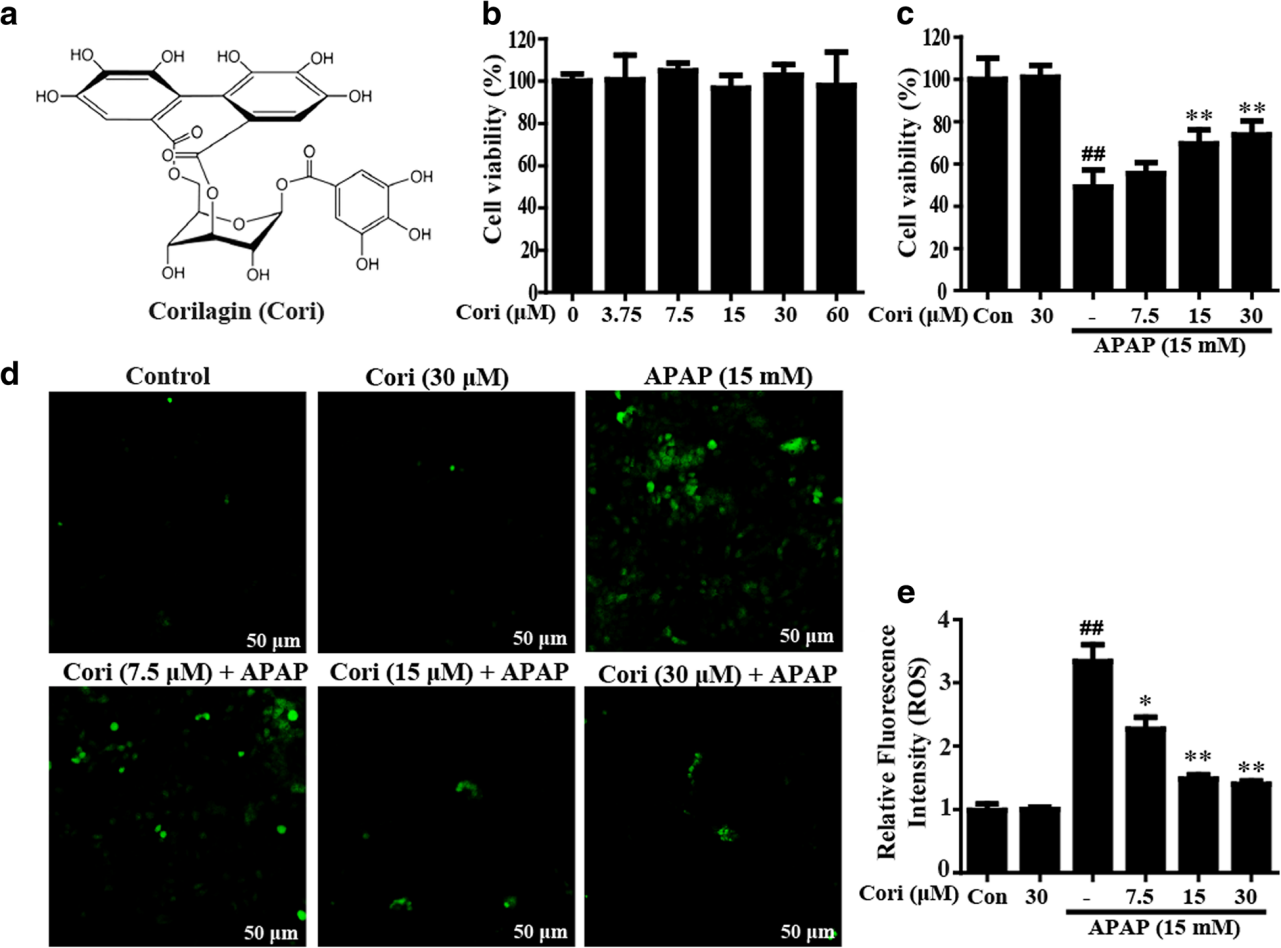
Effect of Cori treatment on APAP-induced hepatotoxicity in HepG2 cells. **a** Chemical structure of corilagin (Cori). **b** HepG2 cells were treated by Cori (0, 3.75, 7.5, 15, 30, or 60 μM) for 24 h. Cell viability was measured by CCK8 assay. **c** Cells were subjected to Cori (7.5, 15 or 30 μM) for 1 h, and then exposed to APAP (15 mM) for 24 h. Cell viability was determined by CCK8 assay. Moreover, (**d**-**e**) the ROS generation was measured according to the Experimental Section and determined by a fluorescent microscope. Similar results were obtained from three independent experiments. All results were expressed as means ± SEM of three independent experiments. ^##^*p* < 0.01 vs the Control group; ^*^p < 0.05, ^**^p < 0.01 vs the APAP group

To date, numerous compounds, including coumarins, flavonoids, quinones and polyphenols, are derived from natural products distributed extensively in vegetables, fruits and widespread medicinal plants. These compounds have been widely thought to be adjuvant and alternative medicines for improvement of hepatic diseases by induction of the Nrf2 signaling pathway [7, 16, 17]. Corilagin (Cori, Fig. 1a), a polyphenol belonging to the tannin family and extracted from *Phyllanthus amarus* and *Geranium carolinianum*, possesses a variety of biological properties, such as antioxidant and anti-inflammatory properties [18–20]. For instance, previous reports have revealed that Cori inhibits the generation of proinflammatory cytokines and reduces ROS production, free radical formation and lipid peroxidation in vitro [21, 22]. Furthermore, Cori has been reported to attenuate lipopolysaccharide (LPS) and D-Galactosamine (GalN)-induced liver failure by suppressing oxidative stress and apoptosis [23]. In the current study, we investigated whether the alleviation of APAP-induced hepatotoxicity by treatment with Cori was correlated with the induction of the AMPK/GSK3β-Nrf2 signaling pathway.

## Methods

### Reagents

Corilagin (Cori), purity up to 98%, was obtained from the Chengdu Herbpurify CO., LTD (Chengdu, China). MTT, DMSO, and Acetaminophen (APAP), were provided by Sigma-Aldrich (St. Louis, MO, USA). DMEM, Penicillin and streptomycin, and Fetal bovine serum (FBS), were offered by Invitrogen-Gibco (Grand Island, NY). Antibodies against p-JNK/JNK, p-AMPK/AMPK, p-ACC/ACC, p-GSK3β Ser ^9^/GSK3β, Keap1, Nrf2, HO-1, GCLC, NQO1, GCLM, Lamin B, β-actin were purchased from Abcam (Cambridge, MA, USA) or Cell Signaling (Boston, MA, USA). Antibodies against p-LBK1/LBK1 were afforded by ABclona Technology (Wuhan, China). Moreover, MPO, MDA, ALT, AST, SOD, GSH and GSSG test kits were supported by Nanjing Jiancheng Bioengineering Institute (Nanjing, China) or Beyotime Biotechnology (Shanghai, China). All other chemicals and reagents were purchased from Sigma-Aldrich (St. Louis, MO, USA).

### CRISPR/Cas9 knockout of Nrf2 gene

The HepG2 hepatocellular carcinoma cells line was offered by the China Cell Line Bank (Beijing China). Briefly, HepG2 cells were knocked off Nrf2 as previously described [24].

### Keap1-siRNA transfection

For Keap1-siRNA transfection, HepG2 cells were seeded into 6-well plates (2 × 10^5^ cells/well) until the confluence of cells approached approximately 60–70%. Next, Keap1-siRNA or Keap1-negative control siRNA (Santa Cruz Biotechnology, Santa Cruz, CA) was transiently transfected into the cells using siRNA transfection reagent lipofectamine™ 2000 (Invitrogen, Carlsbad, CA, USA) according to the manufacturer’s protocol. After 24 h, the transfected cells were treated with Cori for 6 h and followed by lysis buffer for Western blot analysis.

### Cell culture and cytotoxicity

HepG2 cells were maintained with DMEM medium containing 10% of FBS, 100 U/mL of penicillin and streptomycin, as well as 3 mM of glutamine at 37 °C with 5% CO_2_. According to the experimental requirements, the content of cytotoxicity analysis included three parts. The first part, cells were dealt with different concentration of Cori (0, 3.75, 7.5, 15, 30, or 60 μM) for 24 h. The second part, Cells were also pretreated with Cori (7.5, 15 or 30 μM) for 1 h, and then APAP (15 mM) stimulation for 24 h. The third part, WT and Nrf2 ^−/−^ HepG2 cells (1 × 10 ^4^ cells/well) were pre-incubated with Cori (30 μM) for 1 h with or without APAP (15 mM) for 24 h. Lastly, after the indicated treatments, cells were subjected to CCK-8 reagent (Dojindo Molecular Technologies, Dojindo, Japan) at 10 μL/well for 2 h at 37 °C. The optical density (OD) value at 450 nm was detected using a microplate reader.

### Intracellular ROS measurement

The content of ROS from many cells is considered as an indicator that cells are suffered with oxidative stress. In brief, different concentration of Cori (7.5, 15 or 30 μM) exposed to cells for 6 h, and mixed with 50 μM of DCFH-DA for 3 h before APAP (15 mM) stimulation. All of them were assayed using the microplate reader according to the manufacturer’s protocol.

### Preparation of nuclear and cytosolic fractions

According to the manufacturer’s instructions, the nuclear and cytoplasmic extracts were collected by a NE-PER Cytoplasmic and Nuclear Extraction Reagents kit (Pierce Biotechnology, Rockford, IL, USA). All steps must been completed on ice or at 4 °C unless otherwise noted.

### ARE promoter activity

According to the manufacturer’s protocol (Invitrogen, Carlsbad, CA, USA), pGL4.37 and pGL4.74 plasmids were applied to transfect into cells, when they approached approximately 75% confluence. After that, cells were pretreated with Cori in different time points or concentrations, a dual-luciferase reporter assay system (Dual-Glo® Luciferase Assay System) was used to check ARE-driven promoter activity.

### Animals

Male C57BL/6 mice with Nrf2 ^−/−^ (Knockout) and Wild-type (WT), weight 18-22 g, six to eight-week-old, were offered by The Jackson Laboratory (Bar Harbor, ME, USA) and provided by Liaoning Changsheng Technology Industrial Co., LTD (Certificate SCXK2010–0001; Liaoning, China), respectively. These animals were fed for 3 days under SPF-condition. All studies were implemented according to the International Guiding Principles for Biomedical Research Involving Animals, which was stated in the Council for the International Organizations of Medical Sciences.

### Experimental design

The Drug-induced hepatotoxicity was caused by intraperitoneal injection of APAP. To evaluate the protective effects of Cori on APAP-induced hepatotoxicity, WT mice were randomly divided into six groups: the control (PBS), Cori only (60 mg/kg), APAP only (900 mg/kg), Cori (15 mg/kg) + APAP, Cori (30 mg/kg) + APAP and Cori (60 mg/kg) + APAP groups. In brief, mice were administered Cori (15, 30 or 60 mg/kg) intraperitoneally twice (at time intervals of 12 h). After 1 h, mice were challenged with a single dose of APAP (900 mg/kg) or (400 mg/kg). The survival rates of mice were monitored for 48 h after intraperitoneal injection of APAP (900 mg/kg). Moreover, the animals were euthanized and then liver tissues and serum were collected 6 h after APAP (400 mg/kg) administration. On the other side, WT and Nrf2 ^−/−^ mice were applied as described above.

### Histopathological evaluation

Fresh liver tissues were gathered, fixed instantly with formalin, and then embedded in paraffin. Lastly, the liver tissues were cut into a thickness of 5 μm sections, which were stained with hematoxylin-eosin (H & E) staining to assess the liver pathological changes using a light microscopy.

### Biochemical indexes assay

Hepatic tissues and blood of all mice were collected for biochemical analysis. The plasma samples were stood at 37 °C for 30 min. Subsequently centrifugation at 3000 rpm/min for 10 min, the levels of ALT and AST in serum was determined using assay kits. Hepatic homogenates were used for detecting the levels of MPO, GSH-to-GSSG, MDA and SOD levels following by the manufacturer’s instructions.

### Western blotting analysis

Total protein of liver tissues was extracted by the RIPA Lysis Buffer (Beyotime). The concentration of liver protein was detected by the BCA protein assay kit (Thermo). The proteins extracts (20 μg) were loaded onto SDS-10% polyacrylamide gel and transferred to a PVDF membrane. These membranes were immersed into 5% (*w*/*v*) nonfat milk for 2 h. After that, all membranes were probed with many primary antibodies, including p-AMPK, AMPK, p-ACC, ACC, p-LKB1, LKB1, Keap1, Nrf2, p-JNK, JNK, HO-1, GCLC, NQO1, GCLM, p-GSK3β, GSK3β, β-actin and Lamin B, overnight at 4 °C, washed with TBST three times before incubation in HRP-conjugated secondary antibodies (1:3000) at room temperature for 1 h. Finally, the membranes were washed again and observed by the ECL Western blotting detection system. The intensity of the bands was determined by Image J gel analysis software. The above experiments were carried out triplicate.

### Statistical analysis

The results were shown as the means ± SEM and analyzed by SPSS19.0 (IBM). Data from all experimental groups were tested by using one-way ANOVA, whereas multiple comparisons were carried out using the LSD method. *P*-values below 0.05 or 0.01 were thought to statistical significance.

## Results

### Cori inhibited APAP-triggered cytotoxicity and ROS accumulation in HepG2 cells

In the current study, the effect of Cori on cell viability was measured by CCK8 assay. The results suggested that Cori at different concentrations (0–60 μM) did not exhibit toxicity in HepG2 cells (Fig. 1b), implying that Cori did not contribute to cytotoxic effects. Therefore, our work explored the effect of Cori on APAP-triggered cytotoxicity in HepG2 cells. The results of the CCK8 assay also showed that Cori clearly suppressed APAP-stimulated cytotoxicity (Fig. 1c). Given that oxidative stress is a major factor in APAP-induced ALF, we further employed APAP to trigger oxidative injury to explore the antioxidant activity of Cori. Our results found that Cori effectively inhibited APAP-induced ROS overproduction in HepG2 cells, implying that Cori possesses an effective antioxidant property (Fig. 1d, e).

### Cori upregulated the expression of HO-1, NQO1, GCLM, and GCLC in HepG2 cells

**Fig. 2:**
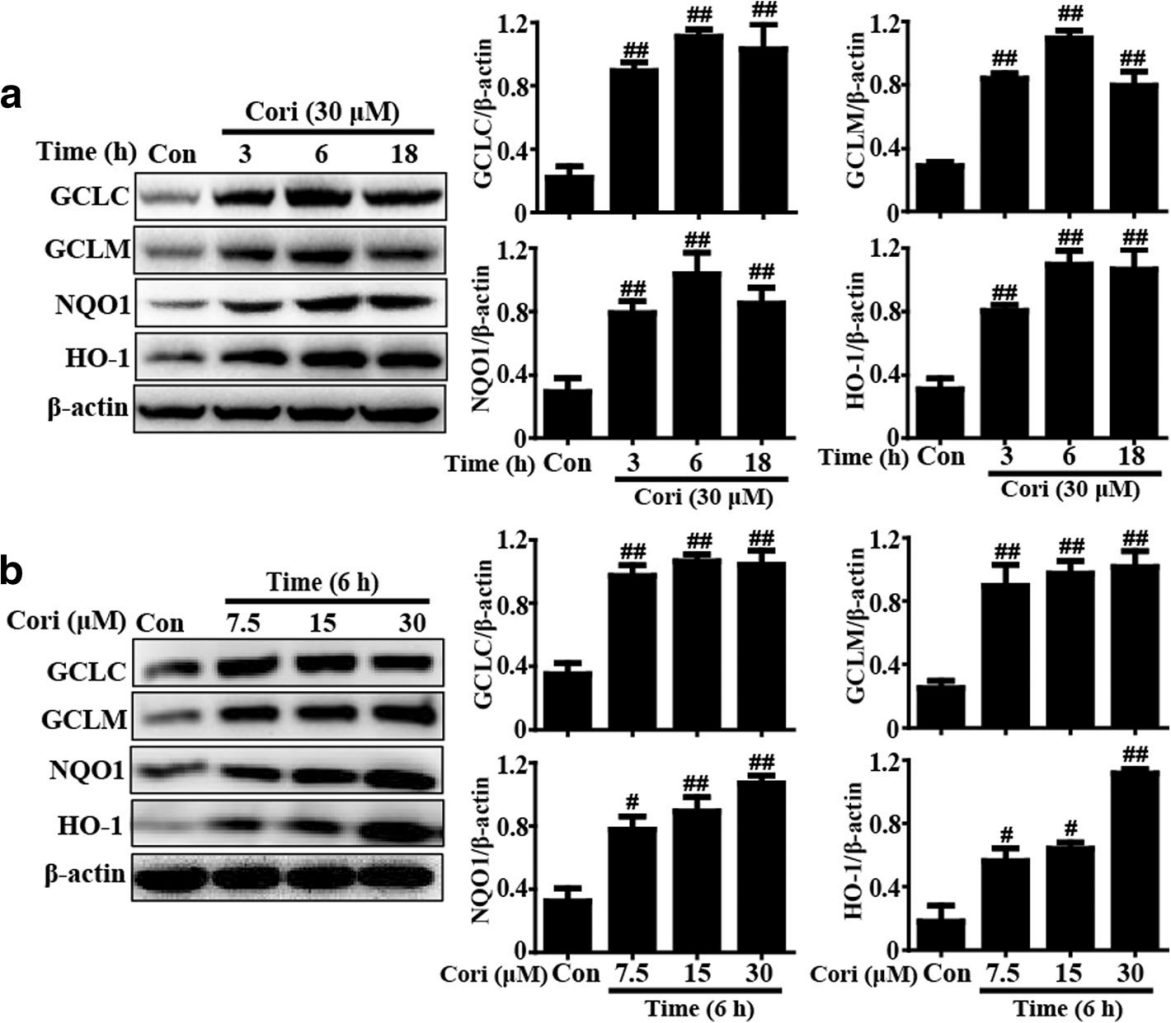
Effects of Cori treatment on GCLC, GCLM, NQO1 and HO-1 in HepG2 cells. **a** HepG2 cells were treated with Cori (30 μM) for three time points (3, 6 or 18 h). In addition, (**b**) Cells were exposed to different concentrations of Cori (7.5, 15, or 30 μM) for 6 h. Protein expressions of GCLC, GCLM, HO-1 and NQO1 were measure by Western blotting analysis. Quantification of GCLC, GCLM, HO-1 and NQO1 protein expressions were performed by densitometric analysis and β-actin was acted as an internal control. Similar results were obtained from three independent experiments. All results were expressed as means ± SEM of three independent experiments. ^#^*p* < 0.05 and ^##^*p* < 0.01 vs the Control group

Because HO-1, NQO1, GCLM, and GCLC are key elements of cellular defense for the improvement of oxidative injury, we explored whether Cori could induce their expression. HepG2 cells were maintained with Cori (30 μM) for three time points to detect the optimal exposure period for upregulating expression of these enzymes. Additionally, the cells were incubated with Cori for 6 h to determine the most effective concentration for enhancing expression of these proteins. Our observations showed that exposure to 30 μM Cori for 6 h dramatically upregulated HO-1, NQO1, GCLM, and GCLC protein expression in HepG2 cells (Fig. 2).

### Cori induced Keap1-Nrf2/ARE signaling pathway activation in HepG2 cells

**Fig. 3:**
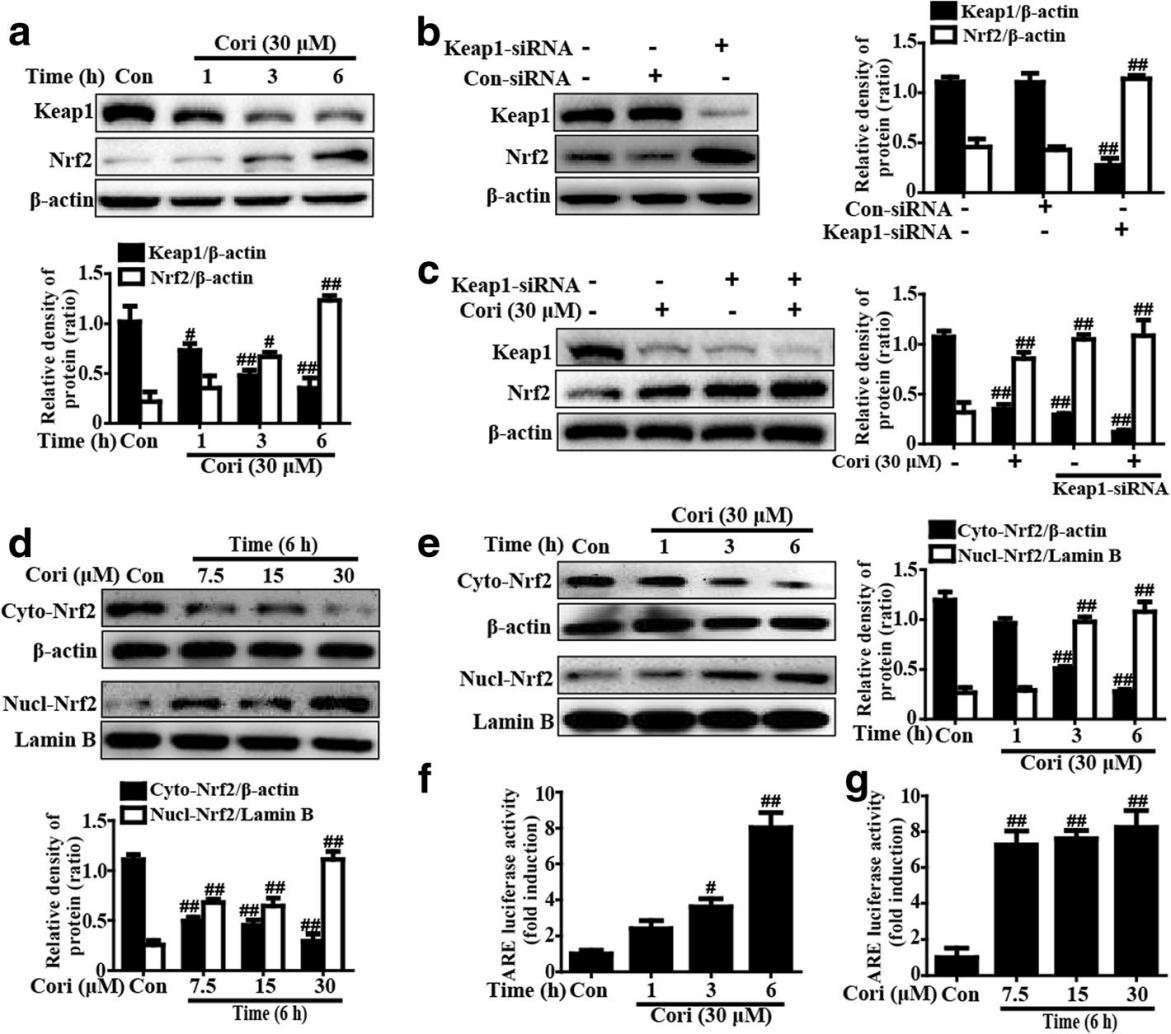
Effects of Cori treatment on Keap1-Nrf2/ARE signaling pathway in HepG2 cells. **a** HepG2 cells were exposed to concentration of Cori (30 μM) for three time points (1, 3 or 6 h), and the total protein were determined by Western blotting analysis. **b** Keap1-siRNA or control siRNA were transfected into cells for 24 h and were collected; proteins were detected by Western blotting analysis. **c** Keap1 mediates Cori-induced Nrf2 protein expression. Keap1-siRNA or control siRNA were transfected into cells for 24 h, then were treated with Cori (30 μM) for 6 h. **d-e** Cells were subjected to Cori (30 μM) for three time points (1, 3 or 6 h) or Cori (7.5, 15, or 30 μM) for 6 h, and the nuclear and cytoplasmic levels of Nrf2 were examined by Western blotting analysis. The relative density of protein was performed by densitometric analysis; β-actin and Lamin B were acted as an internal control. In addition, (**f-g**) the luciferase plasmids pGL-ARE and pRL-TK was transiently transfected into cells for 24 h and subsequently exposed to 30 μM Cori for (1, 3 or 6 h) or Cori (7.5, 15, or 30 μM) for 6 h. ARE luciferase activity was detected by a dual-luciferase reporter assay system. Similar results were obtained from three independent experiments. All results were expressed as means ± SEM of three independent experiments. ^#^*p* < 0.05 and ^##^*p* < 0.01 vs the Control group

Keap1-Nrf2/ARE, an important antioxidant signaling pathway, is required for mediating expression of various antioxidant genes (HO-1, NQO1, GCLM, and GCLC). Thus, we measured whether incubation with Cori could activate the Keap1-Nrf2/ARE signaling pathway. As presented in Fig. 3a, incubation with Cori distinctly increased expression of Nrf2 total protein and degradation of Keap1. To further identify whether the upregulation of Nrf2 by Cori is directly provoked by the inhibition of Keap1, Keap1 siRNA was used to knockdown Keap1. The results demonstrated that the knockdown of Keap1 clearly increased the expression of Nrf2 protein enhanced by Cori (Fig. 3b, c), suggesting that Cori-mediated Nrf2 upregulation is directly associated with the inhibition of Keap1. The results further indicated that Cori significantly decreased the cytoplasmic expression and increased the nuclear accumulation of Nrf2 (Fig. 3d and e). Furthermore, because ARE activation plays an indispensable role in promoting Nrf2 nuclear transcription, Cori-induced enhancement of luciferase activity was accepted as a measure of ARE activation. The results suggested that Cori also efficiently heightened ARE-driven luciferase activity (Fig. 3f and g).

### Cori improved APAP-induced hepatotoxicity through Nrf2 activation in HepG2 cells

**Fig. 4:**
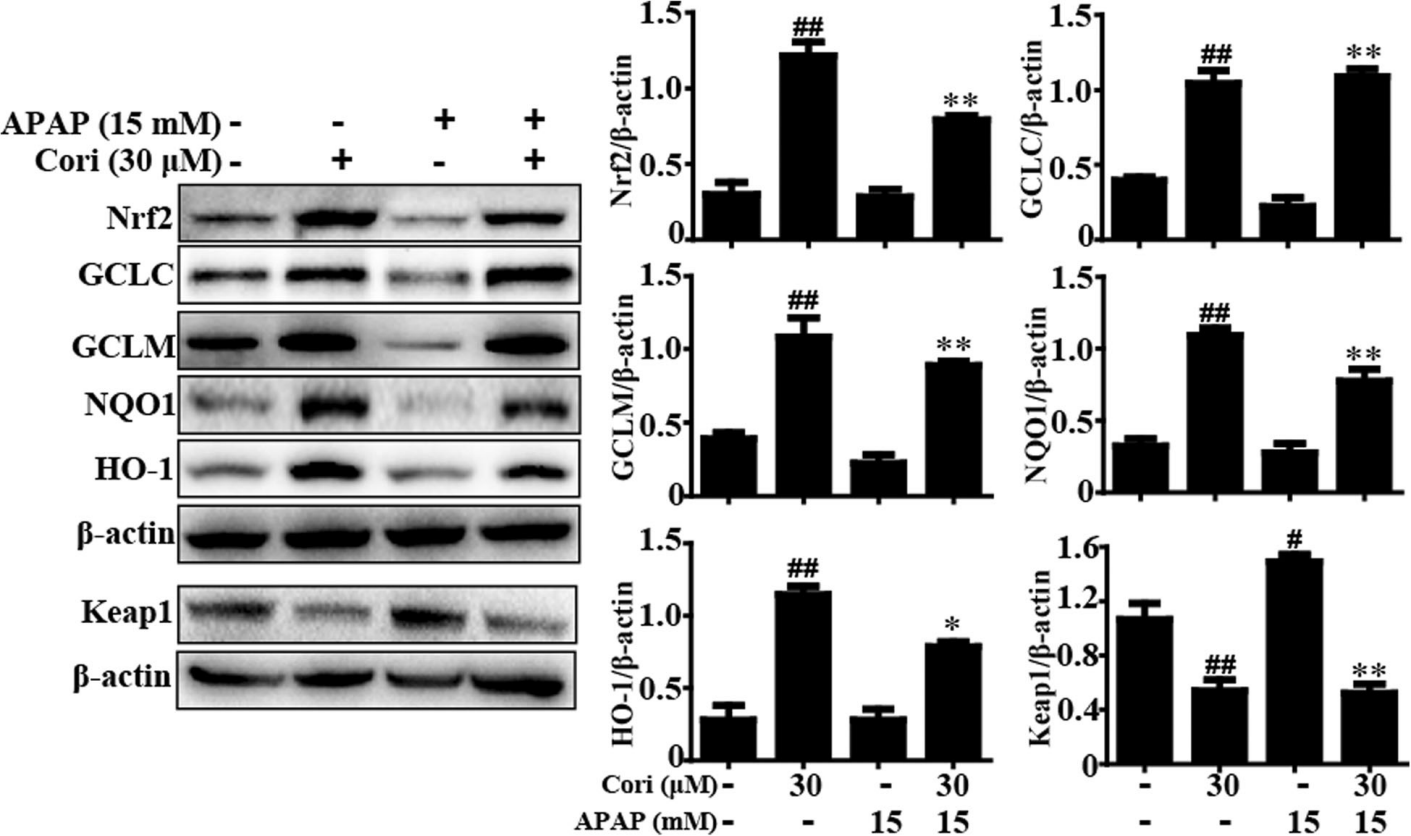
Effect of Cori treatment on Keap1-Nrf2 signaling pathway in HepG2 cells. HepG2 cells were subjected to Cori (30 μM) for 3 h, subsequently stimulated with and without APAP (15 mM) for 3 h. Effects of Cori on Nrf2, GCLC, GCLM, NQO1, HO-1 and Keap1 protein expression. The relative density of protein was performed by densitometric analysis; β-actin was acted as an internal control. Similar results were obtained from three independent experiments. All results were expressed as means ± SEM of three independent experiments. ^#^*p* < 0.05, ^##^*p* < 0.01 vs the Control group; ^*^p < 0.05, ^**^p < 0.01 vs the APAP group

**Fig. 5:**
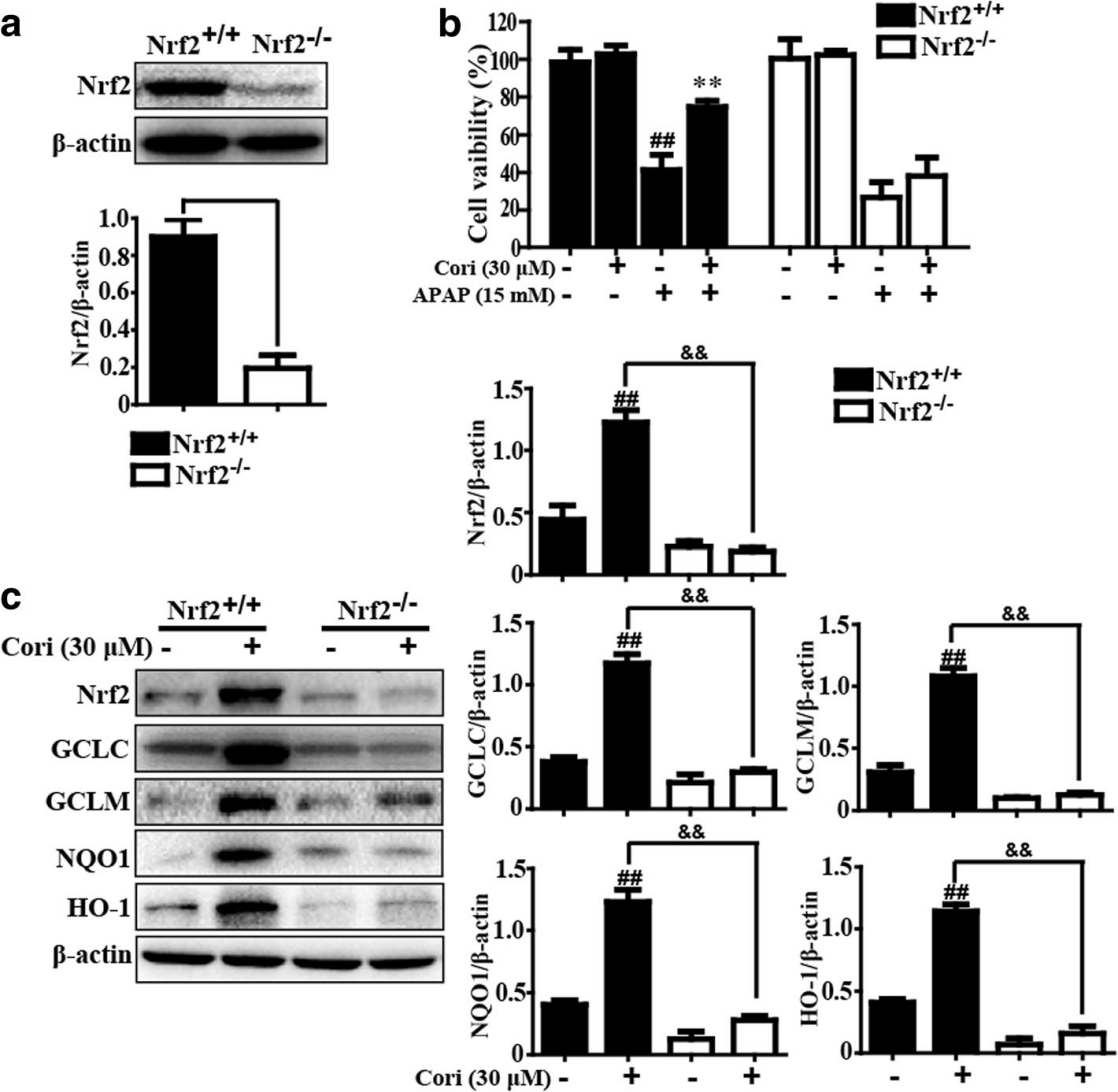
Effects of Cori treatment on Nrf2 signaling pathway in Nrf2 ^−/−^ HepG2 cells. **a** WT and Nrf2^−/−^ HepG2 cells were extracted from total protein for detecting Western blotting analysis. **b** WT and Nrf2 ^−/−^ HepG2 cells were treated with and without Cori (30 μM) for 1 h, and then exposed to APAP (15 mM) for 24 h, cell viability was determined by CCK8 assay. **c** HepG2 WT and Nrf2 ^−/−^ cells were cultured with or without Cori (30 μM) for 6 h, and then immunoblotting was performed to measure Nrf2, GCLC, GCLM, NQO1 and HO-1. The relative density of protein was performed by densitometric analysis; β-actin was acted as an internal control. Similar results were obtained from three independent experiments. All results were expressed as means ± SEM of three independent experiments. ^##^*p* < 0.01 vs the Control group; ^&&^*p* < 0.01 vs the Cori group; ^**^p < 0.01 vs the APAP group

Next, HepG2 cells were pre-incubated with or without Cori and stimulated with APAP, and then Nrf2, Keap1, HO-1, NQO1, GCLM, and GCLC protein expression was analyzed by Western blot. The results revealed that APAP minimally increased the expression of Nrf2, HO-1, NQO1, GCLM, and GCLC proteins but strongly promoted Keap1 protein expression. In contrast, treatment with Cori remarkably enhanced Nrf2, HO-1, NQO1, GCLM, and GCLC expressions and reduced Keap1 expression (Fig. 4). Given these results, we hypothesized that Cori could inhibit hepatotoxicity induced by APAP through Nrf2 activation. To test the hypothesis, the CRISPR/Cas9 gene editing system was used for Nrf2 knockout in HepG2 cells (Fig. 5a). Our results discovered that the inhibition ratio of Cori on APAP-stimulated cell death in HepG2 WT cells was higher than in HepG2 Nrf2 ^−/−^ cells (Fig. 5b). Moreover, the expression of antioxidant genes, such as HO-1, NQO1, GCLM, and GCLC, in WT and Nrf2 ^−/−^ HepG2 cells was determined by Western blot. The data showed that Cori-mediated expression of these proteins was almost blocked in Nrf2 ^−/−^ HepG2 cells (Fig. 5c). These investigations showed that Cori improved APAP-induced hepatotoxicity through Nrf2 activation.

### Cori activated the AMPK/GSK3β signaling pathway in HepG2 cells

**Fig. 6:**
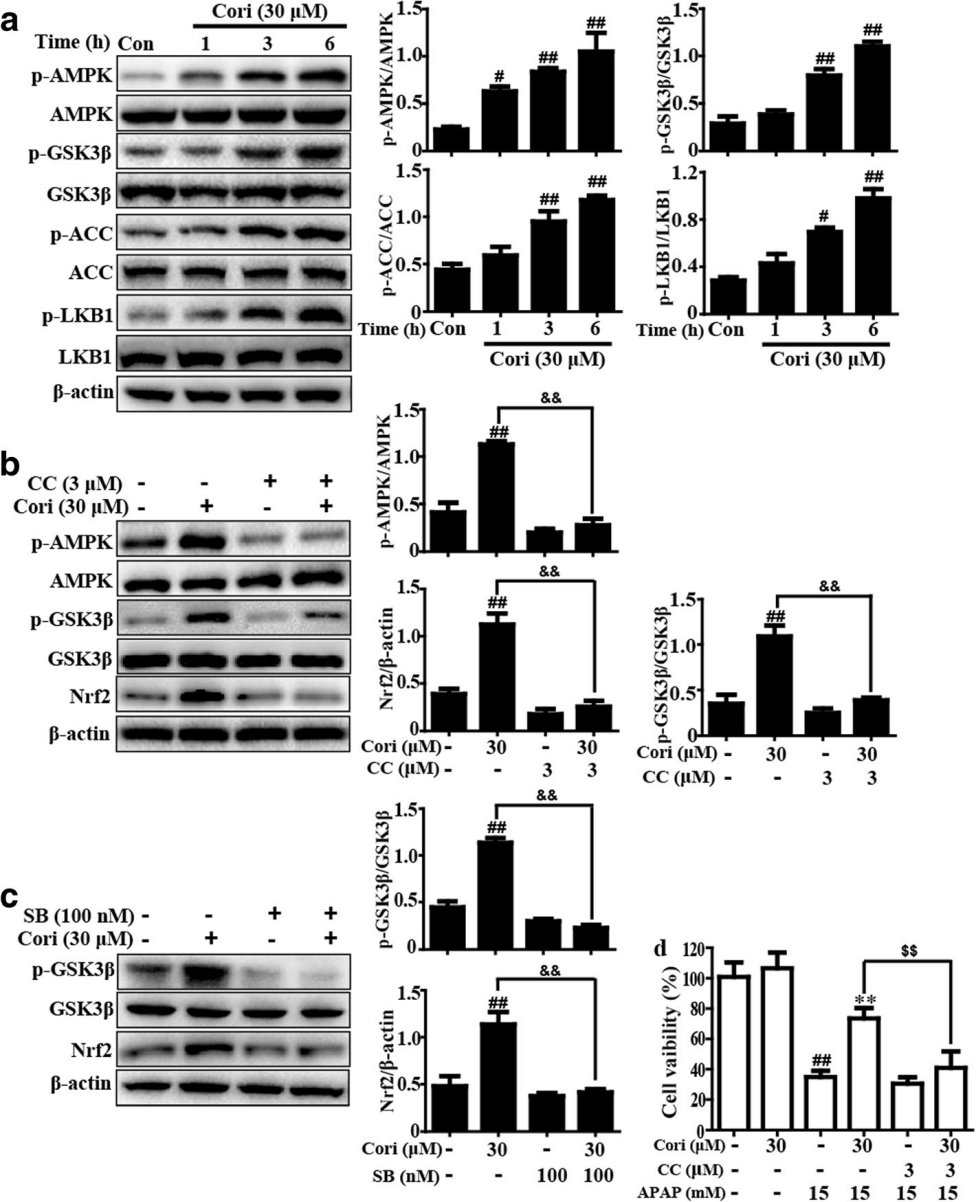
Effects of Cori treatment on AMPK/GSK3β signaling pathway in HepG2 cells. **a** HepG2 cells were exposed to concentration of Cori (30 μM) for three time points (1, 3 or 6 h), and AMPK and GSK3β phosphorylation were determined by Western blotting analysis. **b** Cells were exposed to CC (Compound C: an inhibitor of AMPK, 3 μM) for 18 h and then treated with Cori (30 μM) for 6 h. Effects of Cori on AMPK, GSK3β, ACC and LKB1 phosphorylation and Nrf2 protein expression. Moreover, (**c**) cells were exposed to SB (SB216763: an inhibitor of GSK3β, 100 nM) for 18 h and then treated with Cori (30 μM) for 6 h. Effects of Cori on GSK3β phosphorylation and Nrf2 protein expression. Additionally, (**d**) cells were exposed to CC (3 μM) for 1 h and then treated with Cori (30 μM) for 1 h, and then exposed to APAP (15 mM) for 24 h, cell viability was determined by CCK8 assay. The relative density of protein was performed by densitometric analysis; β-actin was acted as an internal control. Similar results were obtained from three independent experiments. All results were expressed as means ± SEM of three independent experiments. ^#^*p* < 0.05, ^##^*p* < 0.01 vs the Control group; ^**^p < 0.01 vs the APAP group; ^&&^*p* < 0.01 vs the Cori group

To further investigate the upstream regulator of Nrf2 that is induced by Cori, AMPK-mediated GSK3β phosphorylation was detected by Western blot. The results suggested that exposure to 30 μM Cori for 6 h effectively induced phosphorylation of AMPK, GSK3β, ACC and LKB1 in HepG2 cells (Fig. 6a). Additionally, pre-incubation of cells with Compound C (CC, an inhibitor of AMPK, 3 μM) or SB (SB216763: an inhibitor of GSK3β, 100 nM) prevented the Cori-mediated phosphorylation of AMPK and GSK3β, as well as the expression of Nrf2 protein (Fig. 6b and c). Furthermore, cell viability was measured in HepG2 cells that were pretreated with CC (3 μM) for 1 h then exposed to Cori (30 μM) for 6 h and subjected to 15 mM APAP for 24 h. Our results implied that treatment with Cori remarkably decreased cell death compared with treatment with APAP (Fig. 6d). These investigations revealed that Cori-enhanced Nrf2 signaling and cytoprotection may be interrelated in inducing LBK1-AMPK/ACC-GSK3β pathway activation.

### Cori decreased APAP-induced mortality and hepatotoxicity in mice

**Fig. 7:**
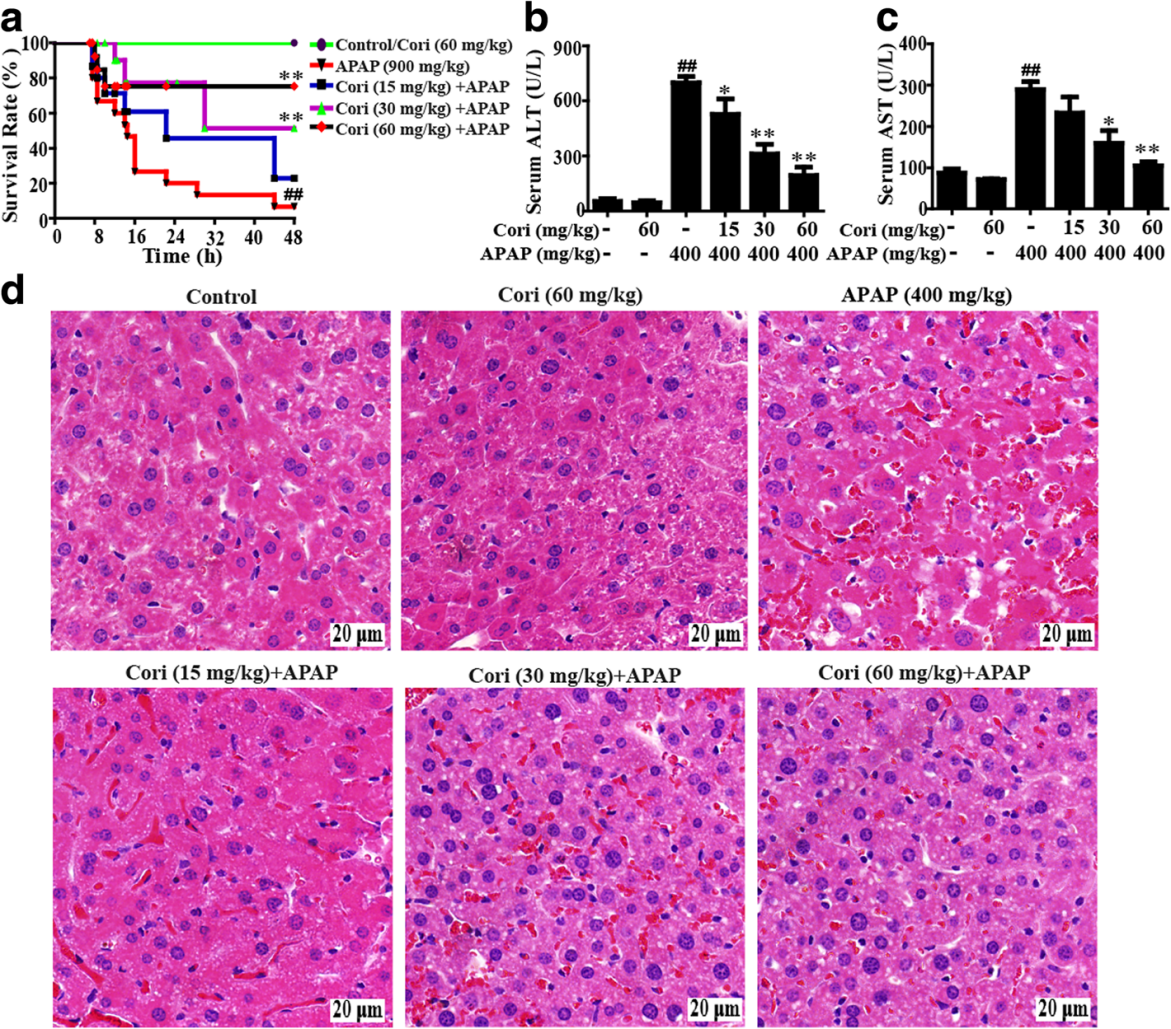
Protective effects of Cori treatment on APAP-induced ALI. **a** Experimental protocol for APAP-induced ALF model, Cori (15, 30 or 60 mg/kg) was administered intraperitoneally to mice for twice at a 12 h (interval for 12 h), followed by subjected treatment with APAP (900 mg/kg). The survival rates of the mice (*n* = 15/group) were observed within 48 h after APAP exposure. **b-c** Sera were collected from the mice after exposure to APAP (400 mg/kg) for 6 h for measurement of the ALT and AST levels. **d** Representative histological sections of the livers were stained with hematoxylin and eosin (H & E)-stained (magnification × 400). Similar results were obtained from three independent experiments. All data are presented as means ± SEM (*n* = 5/group). ^*##*^*p* < 0.01 vs. Control group; ^*^p < 0.05*,* ^**^p < 0.01 vs. APAP group

Based on the results above, we further explored whether Cori could mitigate APAP-induced hepatotoxicity by detecting the mortality rate of mice within 48 h after APAP challenge. As presented in Fig. 7a, the mice died 7 h after APAP injection, and the mortality rate reached 95% at 48 h, whereas pre-injection with Cori, especially at 60 mg/kg), effectively reduced the mortality rate to approximately 30%. Moreover, since plasma ALT and AST levels are considered crucial markers of hepatic injury, ALT and AST serum levels in mice with ALF were examined. Our results found that ALT and AST serum levels were noticeably elevated at 6 h after APAP challenge. However, this increase was significantly reduced by treatment with Cori, suggesting that treatment with Cori effectively protected against APAP-induced ALF (Fig. 7b and c). Next, the effects of Cori on APAP-induced liver histopathological changes were observed by H & E staining. In this study, the APAP group displayed apparent disturbed architecture, including hepatocyte necrosis, hemorrhage and neutrophil infiltration, which were remarkably relieved by treatment with Cori (Fig. 7d).

### Cori ameliorated APAP-induced oxidative injury in mice with ALF

**Fig. 8:**
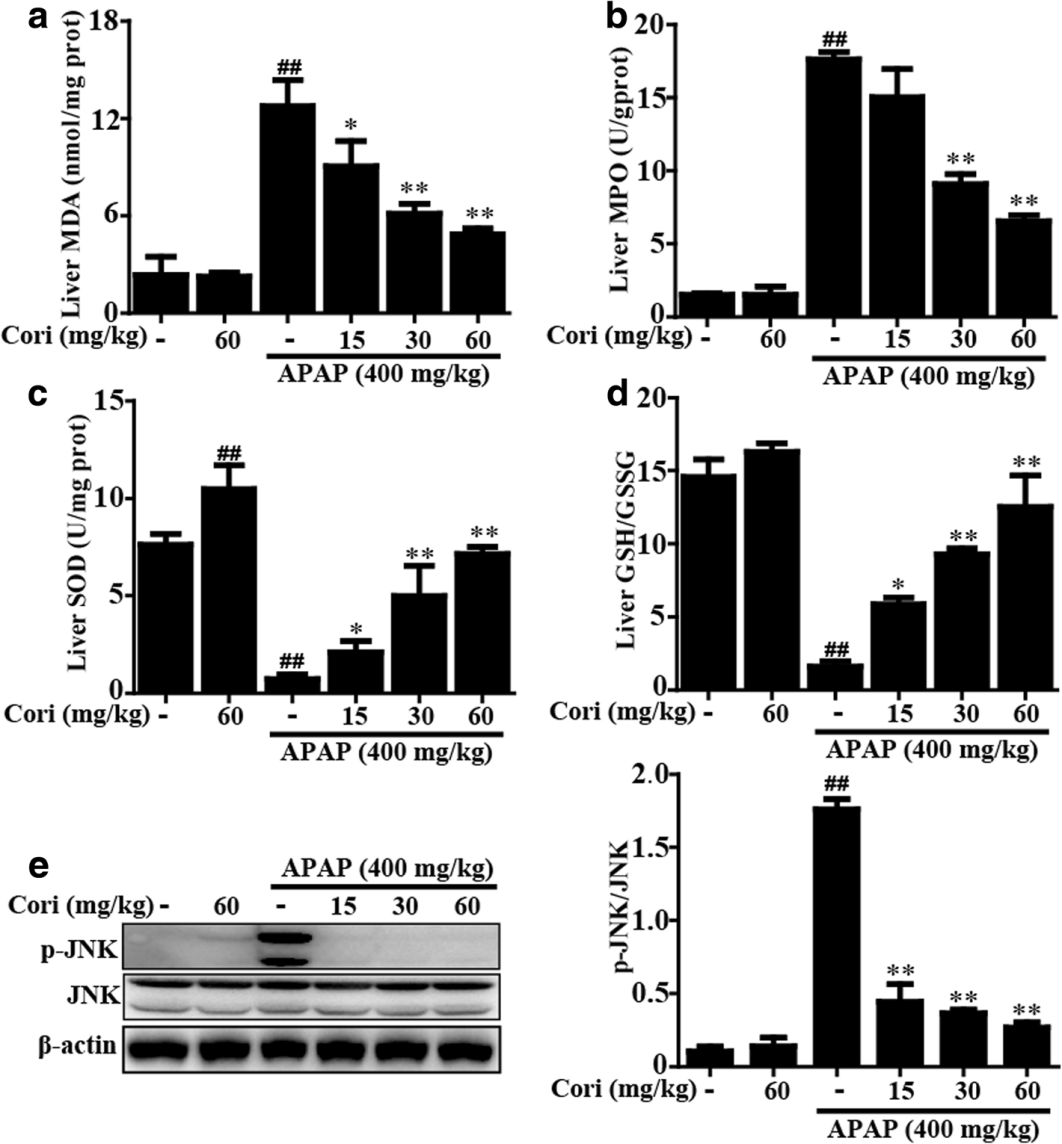
Effect of Cori treatment on APAP-induced oxidative damage. **a-d** Liver tissues were collected from the mice 6 h after APAP challenged for measurement of SOD depletion, MPO and MDA formation, and GSH-to-GSSG levels. **e** The effects of Cori on APAP-induced JNK phosphorylation were analyzed by Western blotting analysis. Quantification of relative protein expression was performed by densitometric analysis. Similar results were obtained from three independent experiments. All data are presented as means ± SEM (*n* = 5/group). ^##^*p* < 0.01 vs. Control group; ^*^p < 0.05, ^**^p < 0.01 vs. APAP group

Oxidative injury is one of the key characteristics found in mice with APAP-induced ALF. Therefore, we investigated whether Cori could protect against hepatotoxicity by inhibiting oxidative stress. Our results determined that APAP not only significantly reduced SOD depletion but also clearly promoted MDA and MPO formation in the liver of mice, whereas pretreatment with Cori markedly blocked these effects (Fig. 8a-c). Additionally, GSH-to-GSSG ratio was measured to evaluate the generation of ROS, and this ratio was noticeably increased in mice pretreated with Cori when compared to APAP injection only (Fig. 8d). Furthermore, several researchers have previously noted that JNK activation, which is related to APAP hepatotoxicity, also amplified ROS formation. As shown in Fig. 8, the phosphorylation of JNK was remarkably increased by APAP overdose, whereas the effect was clearly suppressed by pretreatment with Cori.

### Cori enhanced AMPK/GSK3β-Nrf2 signaling in APAP-induced ALF

**Fig. 9:**
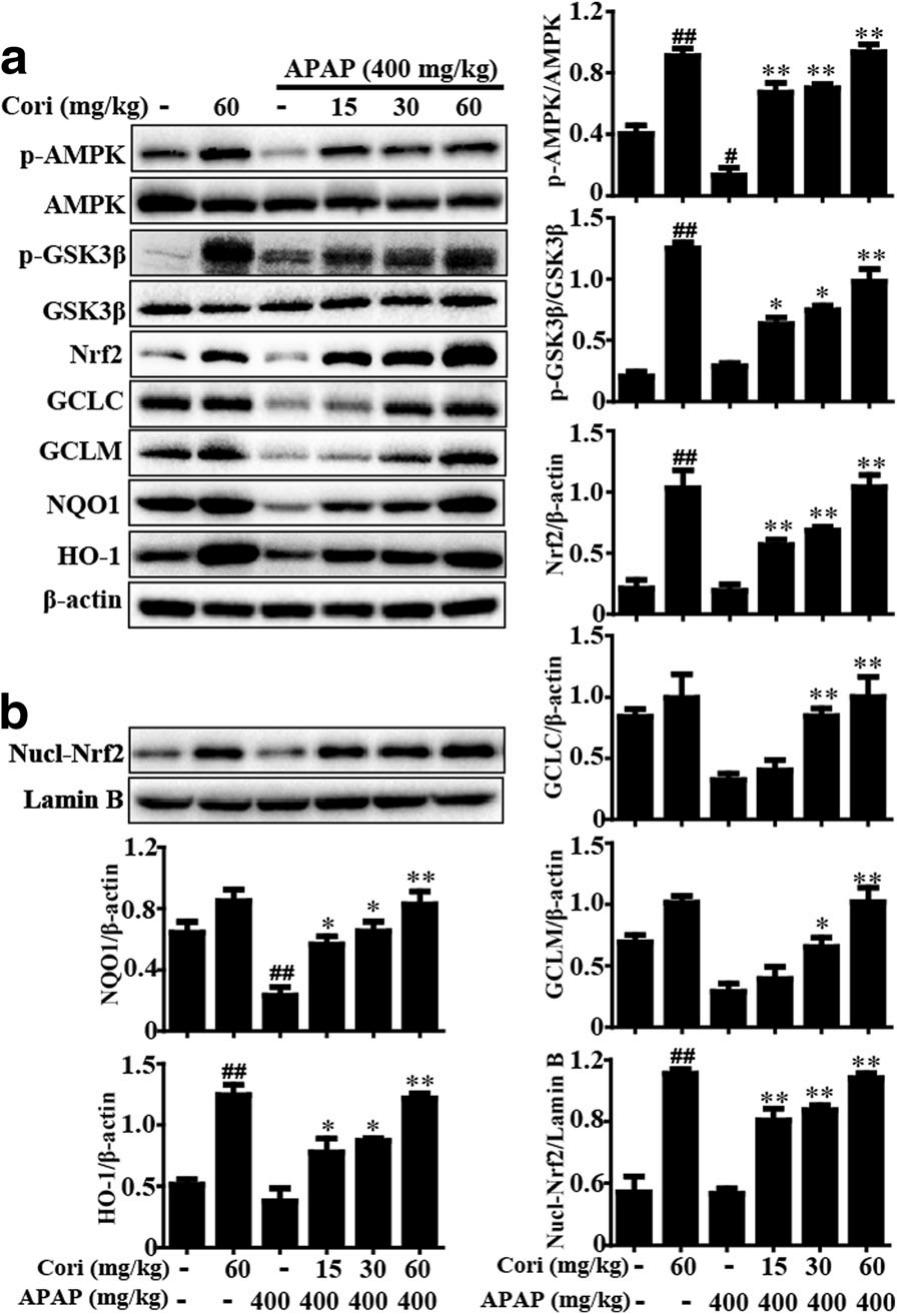
Effects of Cori on AMPK/GSK3β and Nrf2 pathway in APAP-induced ALF. Liver tissues were collected from the mice 6 h after APAP challenge for Western blotting analysis. **a** The effects of Cori on p-AMPK, p-GSK3β, Nrf2, GCLC, GCLM, HO-1 and NQO1 protein expression. **b** The effects of Cori on Nrf2 nuclear translocation. Quantification of relative protein expression was performed by densitometric analysis. Similar results were obtained from three independent experiments. All data are presented as means ± SEM (*n* = 5/group). ^*##*^*p* < 0.01 vs. Control group; ^*^p Cori reveiwers*,* ^**^p < 0.01 vs. APAP group

To further investigate the hepatoprotective mechanism of Cori against APAP-induced ALF in mice, we explored the effects of Cori on the activities of AMPK, GSK3β, Nrf2, HO-1, NQO1, GCLM, and GCLC. As shown in Fig. 9, pretreatment with Cori not only phosphorylated AMPK and GSK3β but also promoted the induction of Nrf2, HO-1, NQO1, GCLM, and GCLC protein expression in liver tissue. Moreover, our findings further revealed that pretreatment with Cori could induce an increase in the nuclear levels of Nrf2 in APAP-induced ALF. These results are consistent with our in vitro results.

### Cori relieved APAP-induced ALF through Nrf2 activation

**Fig. 10:**
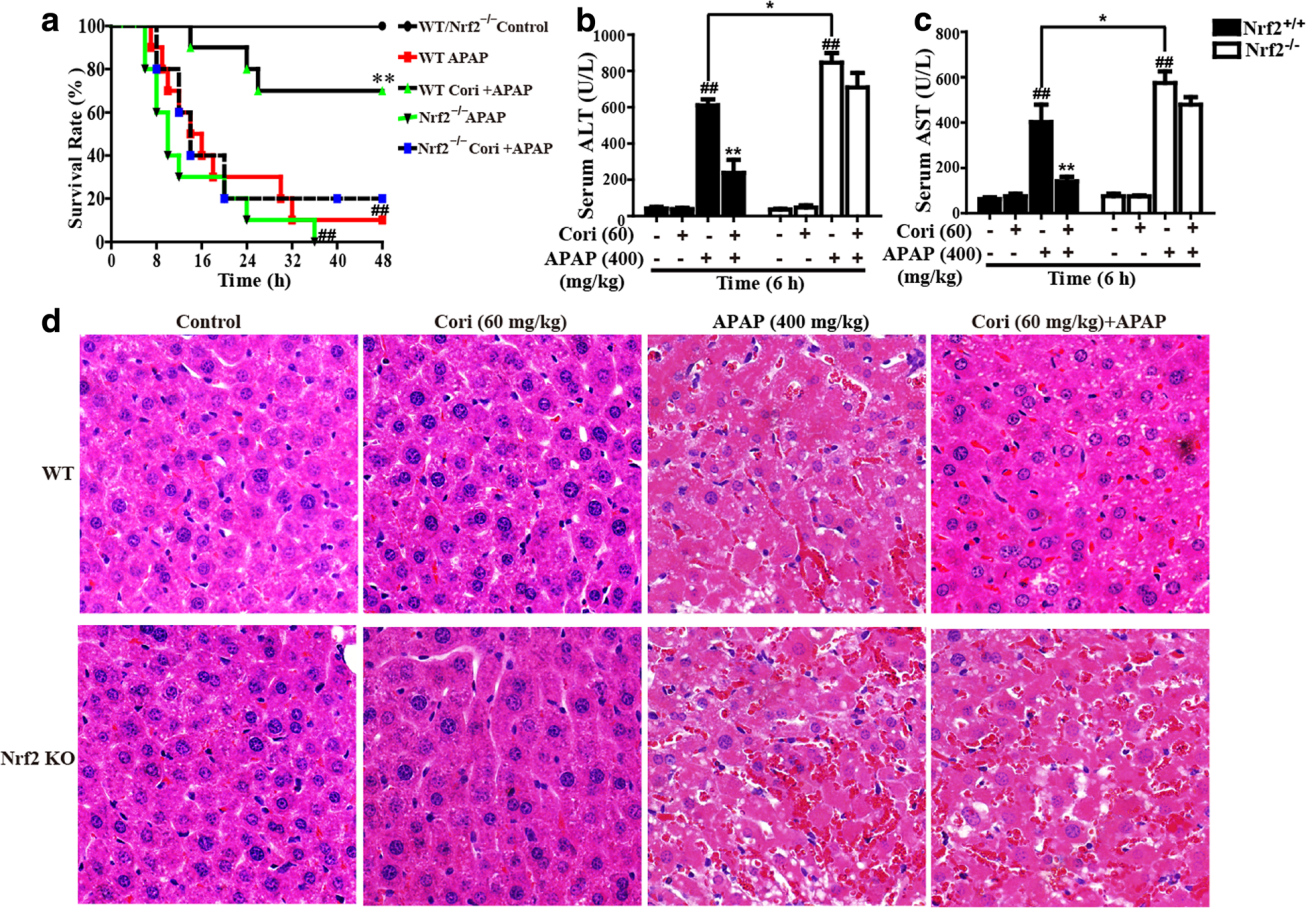
Protective effects of Cori-meditated Nrf2 on APAP-induced mice with ALF. WT and Nrf2 ^−/−^ mice (n = 15 /group) were intraperitoneally injected Cori (60 mg/kg) with mice for twice at a 12 h (interval for 12 h), followed by subjected treatment with APAP (900 mg/kg). **a** The survival rates of the mice were observed within 48 h after APAP exposure. Moreover, (**b-c**) serum was collected from the mice after exposure to APAP (400 mg/kg) for 6 h for measurement of the ALT and AST levels. (**d**) Livers (*n* = 5) from each experimental group were processed for histological evaluation at 6 h after the APAP (400 mg/kg) challenge. Similar results were obtained from three independent experiments. All data are presented as means ± SEM (*n *= 5/group). ^##^*p* < 0.01 vs. Control group; ^*^p < 0.05, ^**^p < 0.01 vs. APAP group

Last but not least, our studies further clarified whether Cori could attenuate APAP-induced ALF through Nrf2 upregulation by observing the effect of Cori on the survival rate of WT and Nrf2 ^−/−^ mice. As presented in Fig. 10a, pretreatment with Cori increased the survival rate of APAP-induced WT mice, which declined from approximately 70 to 20% in Nrf2 ^−/−^ mice. Our results suggested that Cori-induced inhibition of ALT and AST plasma levels in WT mice was effectively impeded in Nrf2 ^−/−^ mice (Fig. 10b and c). Furthermore, we observed histopathological changes in WT and Nrf2 ^−/−^ mice, showing that treatment with Cori relieved serious histopathological changes in WT mice and these changes were dramatically eliminated in Nrf2 ^−/−^ mice (Fig. 10d). These investigations illustrated that Cori-mediated hepatoprotective effects may be associated with Nrf2 pathway activation.

## Discussion

Acute liver failure (ALF) resulting from APAP overdose, which is among the most well-studied drug-induced diseases, is a severe clinical condition that is mostly caused by oxidative stress and starts with uncontrolled ROS accumulation and can ultimately result in death [2, 25]. It is reported that the APAP overdose-induced model in rodents is universally accepted as an experimental in vivo system for exploring novel therapeutic strategies, including the testing of natural products [26]. Intriguingly, several previous studies have indicated that Nrf2, a critical coordinator for oxidative stress that can be activated by natural products, functioned efficiently in the alleviation of liver injury [26, 27]. Corilagin (Cori), which is a natural polyphenol compound, exhibited antioxidant and anti-inflammatory activities and attenuated GalN/LPS-sensitized hepatic failure and suppression of oxidative stress and apoptosis [22, 23]. In the current study, we discovered that Cori could protect against APAP-induced ALF through the induction of Nrf2 via the AMPK/GSK3β pathway.

Hepatotoxicity induced by APAP is a complex course that includes the generation of oxidative stress, inflammation, hepatocyte cell death, and regeneration. Oxidative stress has long been recognized to be responsible for the occurrence and progression of APAP hepatotoxicity [25]. Therefore, it is important to explore if Cori can suppress oxidative stress. Our findings clearly indicated that Cori reduced APAP-triggered ROS overproduction and cytotoxicity in HepG2 cells, supporting the finding that Cori inhibited oxidative stress. Furthermore, the elimination of ROS involves generation of antioxidant enzymes, including HO-1, NQO1, GCLM, and GCLC, which are considered an important component of the cellular defense mechanism towards oxidative stress [28, 29]. In this study, treatment with Cori remarkably induced protein expression of antioxidant enzymes HO-1, NQO1, GCLM, and GCLC in HepG2 cells. Importantly, Nrf2, as a coordinator for many signaling pathways, mitigates oxidative stress-induced cell injury via mediating expression of a variety of genes [30, 31]. Our results suggested that Cori clearly upregulated the Keap1-Nrf2/ARE signaling pathway. Further, Cori-mediated cytoprotection was impeded in Nrf2 ^−/−^ HepG2 cells, indicating that Nrf2 modulated Cori protection against APAP toxicity. Furthermore, antioxidant enzymes, such as HO-1, NQO1, GCLM, and GCLC, are refractory to Cori-mediated overexpression in Nrf2 ^−/−^ HepG2 cells. Thus, Cori protected against APAP toxicity and promoted expression of antioxidant enzymes, which is dependent on Nrf2. More importantly, numerous studies have previously revealed that AMPK results in an increase in Nrf2 nuclear accumulation by phosphorylation in addition to GSK3β inhibition [11, 32]. In the current study, treatment with Cori could effectively induce the phosphorylation of AMPK and GSK3β in HepG2 cells. Further, the addition of inhibitors found that Cori-mediated Nrf2 pathway activation may rely on the regulation of AMPK/GSK3β. Additionally, considering that LKB1 and ACC are a key upstream kinase and critical downstream molecule of AMPK, respectively, and are essential for its activation [33, 34], our results further noticed that the increases in the phosphorylation of LBK1 and ACC were induced by treatment with Cori. Furthermore, cell viability decreased significantly when treatment with Cori was combined with AMPK inhibitors in APAP-induced HepG2 cells. These results demonstrated that treatment with Cori inhibited APAP-induced hepatotoxicity via its upregulation of the AMPK/GSK3β-Nrf2 signaling pathway.

Based on the in vitro results above, we conducted further studies to investigate the protective effect of Cori on APAP-induced ALF in mice and the underlying mechanism. Serum ALT and AST levels, which are markers of liver injury severity, indicate the possible pathological status in liver [35]. In this study, treatment with Cori significantly reduced serum levels of ALT and AST and reduced APAP-induced mortality. Moreover, results of histopathological examination showed that treatment with Cori effectively relieved liver histopathological changes. On the one hand, it is reported that administration of APAP to mice increases the formation of MPO and MDA as well as the depletion of superoxide dismutase (SOD), aggravating liver tissue damage [36]. Moreover, members of the glutathione pool, GSH-to-GSSG, are essential in many cells as a key buffer to maintain cellular redox status, and alteration of their ratios possibly indicates intracellular redox homeostasis [37], which is crucial for the improvement of APAP-induced ALF [38]. In this work, treatment with Cori dramatically reduced the formation of MDA and MPO, increased depletion of SOD, and decreased the GSH to GSSG ratio, indicating that treatment with Cori significantly relieved oxidative injury induced by APAP. On the other hand, JNK activation mediated by oxidative stress is also vital to APAP-induced hepatic injury [39, 40]. Our study clearly indicated that Cori could inhibit JNK activation in mice with ALF. These investigations indicated that Cori effectively improved APAP-induced oxidative damage in mice. Interestingly, Nrf2 signaling has been thought to be a potential target for new therapeutics in liver disease [41]. In addition, recent studies have reported that enhanced Nrf2 antioxidant signaling is essential for protecting against liver injury induced by APAP [42]. Accordingly, given our result that Cori inhibited APAP-induced hepatotoxicity by Nrf2 upregulation in vitro, we further measured the effect of Cori on the Nrf2 signaling pathway in mice with APAP-induced ALF. The results suggested that treatment with Cori significantly raised the protein expression levels of Nrf2, HO-1, NQO1, GCLM, and GCLC in an APAP-induced ALF mouse model. To further demonstrate the role of Nrf2 upregulation in Cori-mediated alleviation of liver injury caused by APAP, Nrf2-deficienct mice were used in the study. The APAP-induced mortality in WT mice that was reduced by Cori was markedly blocked in Nrf2 ^−/−^ mice. More crucially, recent reports have indicated that Nrf2 transcription exerts potential hepatoprotective effects related to the activation of the AMPK/GSK3β pathway in a model of liver injury [43, 44]. Our findings unveiled that Cori pretreatment clearly promoted the phosphorylation of AMPK and GSK3β in mice with ALF. In general, our experimental results provide substantial evidence that Cori is crucial for the prevention of APAP-induced hepatic injury via upregulation of the Nrf2 signaling pathway, which might rely on the phosphorylation of AMPK and GSK3β to a fairly great extent.

## Conclusions

**Fig. 11:**
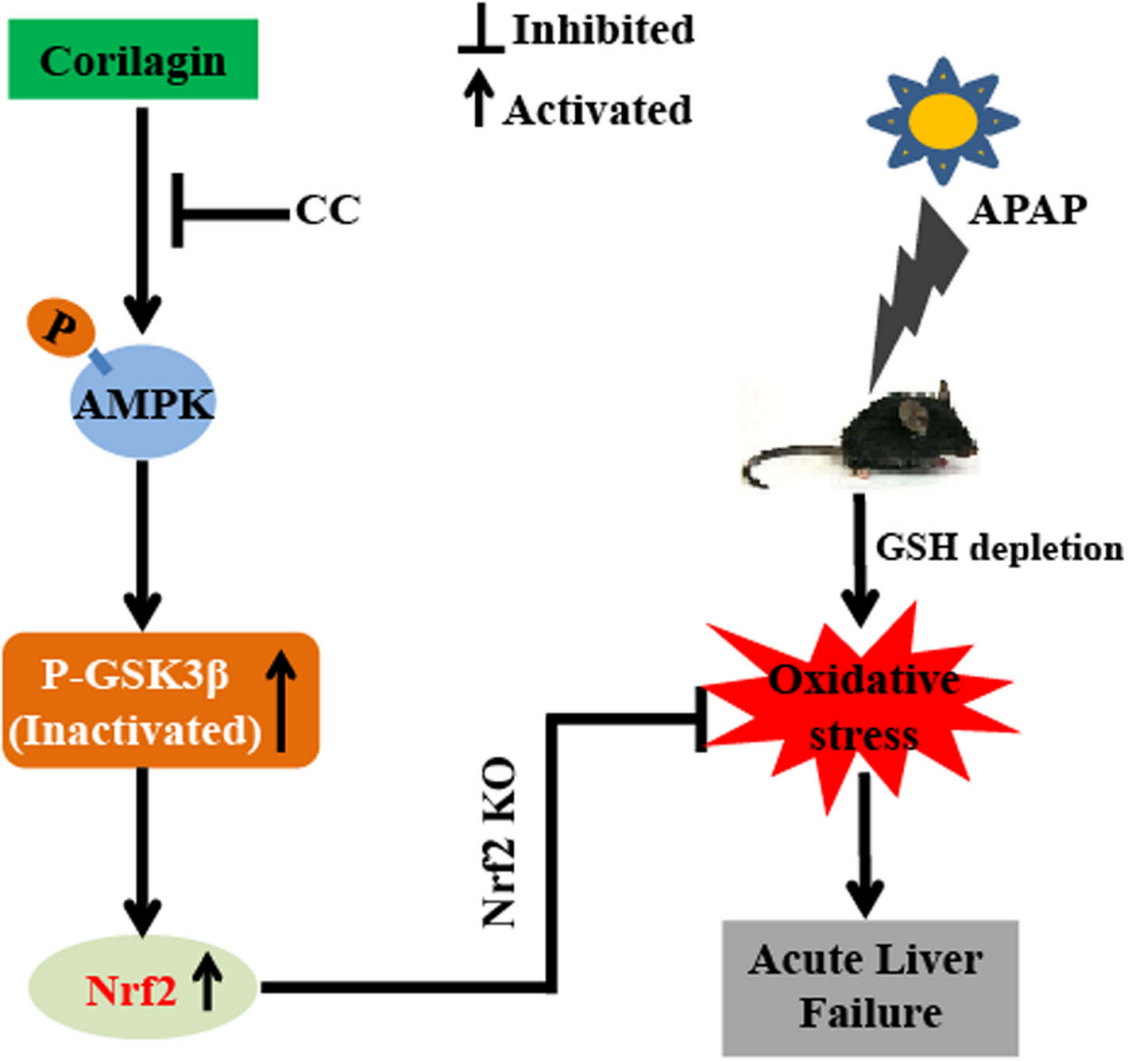
Cori-mediated Nrf2 defense protects APAP-induced acute liver failure via induction of AMPK/GSK3β pathway

In conclusion, as shown in Fig. 11, this study suggests that Cori has a protective effect on liver injury resulting from APAP. The underlying mechanisms may be closely associated with the evident induction of HO-1, NQO1, GCLM, and GCLC, and the increase in the GSH-to-GSSG ratio, which may rely on Cori-mediated Nrf2 defense mechanisms in an AMPK/GSK3β dependent manner. Hence, our study gives evidence of benefit for the application of Cori in protecting against APAP-induced ALF.

## Abbreviations

(CCK-8): Cell counting kit-8
ACC: Acetyl-CoA carboxylase
ALF: Acute liver failure
ALT: Alanine transaminase
AMPK: AMP-activated protein kinase
APAP: Acetaminophen
ARE: Antioxidant response element
AST: Aspartate aminotransferase
Cori: Corilagin
DILI: Drug-induced liver injury
DMSO: Dimethyl sulfoxide
GCLC/GCLM: Glutamate-cysteine ligase catalytic/modifier
GSH: Glutathione
GSK3β: Glycogen synthase kinase-3β
HO-1: Heme oxygenase-1
JNK: c-jun N-terminal kinase
Keap1: Kelch-like ECH-associated protein 1
LKB1: Liver kinase B1
MDA: Malondialdehyde
MPO: Myeloperoxidase
NAPQI: N-acetyl-p-benzoquinone imine
NQO1: NAD (P) H: quinone oxidoreductase
Nrf2: Nuclear factor-erythroid 2-related factor 2
ROS: Reactive oxygen species
SOD: Superoxide dismutase

## References

[1] Bunchorntavakul C, Reddy KR (2013). Acetaminophen-related hepatotoxicity. Clin Liver Dis.

[2] Larson AM, Polson J, Fontana RJ, Davern TJ, Lalani E, Hynan LS, Reisch JS, Schiodt FV, Ostapowicz G, Shakil AO (2005). Acetaminophen-induced acute liver failure: results of a United States multicenter, prospective study. Hepatology.

[3] Jaeschke H (2015). Acetaminophen: dose-dependent drug hepatotoxicity and acute liver failure in patients. Dig Dis.

[4] Larson AM (2007). Acetaminophen hepatotoxicity. Clin Liver Dis.

[5] Jadeja RN, Urrunaga NH, Dash S, Khurana S, Saxena NK (2015). Withaferin-a reduces acetaminophen-induced liver injury in mice. Biochem Pharmacol.

[6] Saberi B, Ybanez MD, Johnson HS, Gaarde WA, Han D, Kaplowitz N (2014). Protein kinase C (PKC) participates in acetaminophen hepatotoxicity through c-Jun-N-terminal kinase (JNK)-dependent and -independent signaling pathways. Hepatology.

[7] Kumar H, Kim IS, More SV, Kim BW, Choi DK (2014). Natural product-derived pharmacological modulators of Nrf2/ARE pathway for chronic diseases. Nat Prod Rep.

[8] Xu D, Chen L, Chen X, Wen Y, Yu C, Yao J, Wu H, Wang X, Xia Q, Kong X (2017). The triterpenoid CDDO-imidazolide ameliorates mouse liver ischemia-reperfusion injury through activating the Nrf2/HO-1 pathway enhanced autophagy. Cell Death Dis.

[9] Malhotra D, Portales-Casamar E, Singh A, Srivastava S, Arenillas D, Happel C, Shyr C, Wakabayashi N, Kensler TW, Wasserman WW (2010). Global mapping of binding sites for Nrf2 identifies novel targets in cell survival response through ChIP-Seq profiling and network analysis. Nucleic Acids Res.

[10] Niso-Santano M, Gonzalez-Polo RA, Bravo-San Pedro JM, Gomez-Sanchez R, Lastres-Becker I, Ortiz-Ortiz MA, Soler G, Moran JM, Cuadrado A, Fuentes JM (2010). Activation of apoptosis signal-regulating kinase 1 is a key factor in paraquat-induced cell death: modulation by the Nrf2/Trx axis. Free Radic Biol Med.

[11] Joo MS, Kim WD, Lee KY, Kim JH, Koo JH, Kim SG (2016). AMPK facilitates nuclear accumulation of Nrf2 by phosphorylating at serine 550. Mol Cell Biol.

[12] Zong H, Ren JM, Young LH, Pypaert M, Mu J, Birnbaum MJ, Shulman GI (2002). AMP kinase is required for mitochondrial biogenesis in skeletal muscle in response to chronic energy deprivation. Proc Natl Acad Sci U S A.

[13] Wu W, Wang S, Liu Q, Wang X, Shan T, Wang Y (2018). Cathelicidin-WA attenuates LPS-induced inflammation and redox imbalance through activation of AMPK signaling. Free Radic Biol Med.

[14] Wang L, Zhang S, Cheng H, Lv H, Cheng G, Ci X (2016). Nrf2-mediated liver protection by esculentoside a against acetaminophen toxicity through the AMPK/Akt/GSK3beta pathway. Free Radic Biol Med.

[15] Bhushan B, Poudel S, Manley MW, Roy N, Apte U (2017). Inhibition of glycogen synthase kinase 3 accelerated liver regeneration after acetaminophen-induced hepatotoxicity in mice. Am J Pathol.

[16] Gao B, Doan A, Hybertson BM (2014). The clinical potential of influencing Nrf2 signaling in degenerative and immunological disorders. Clin Pharmacol Adv Appl.

[17] Iranshahy M, Iranshahi M, Abtahi SR, Karimi G (2018). The role of nuclear factor erythroid 2-related factor 2 in hepatoprotective activity of natural products: a review. Food Chem Toxicol.

[18] Muresan XM, Cervellati F, Sticozzi C, Belmonte G, Chui CH, Lampronti I, Borgatti M, Gambari R, Valacchi G (2015). The loss of cellular junctions in epithelial lung cells induced by cigarette smoke is attenuated by corilagin. Oxidative Med Cell Longev.

[19] Sudjaroen Y, Hull WE, Erben G, Wurtele G, Changbumrung S, Ulrich CM, Owen RW (2012). Isolation and characterization of ellagitannins as the major polyphenolic components of Longan (Dimocarpus longan Lour) seeds. Phytochemistry.

[20] Jin F, Cheng D, Tao JY, Zhang SL, Pang R, Guo YJ, Ye P, Dong JH, Zhao L (2013). Anti-inflammatory and anti-oxidative effects of corilagin in a rat model of acute cholestasis. BMC Gastroenterol.

[21] Zhao L, Zhang SL, Tao JY, Pang R, Jin F, Guo YJ, Dong JH, Ye P, Zhao HY, Zheng GH (2008). Preliminary exploration on anti-inflammatory mechanism of Corilagin (beta-1-O-galloyl-3,6-(R)-hexahydroxydiphenoyl-D-glucose) in vitro. Int Immunopharmacol.

[22] Chen Y, Chen C (2011). Corilagin prevents tert-butyl hydroperoxide-induced oxidative stress injury in cultured N9 murine microglia cells. Neurochem Int.

[23] Kinoshita S, Inoue Y, Nakama S, Ichiba T, Aniya Y (2007). Antioxidant and hepatoprotective actions of medicinal herb, Terminalia catappa L. from Okinawa Island and its tannin corilagin. Phytomedicine.

[24] Qi Z, Ci X, Huang J, Liu Q, Yu Q, Zhou J, Deng X (2017). Asiatic acid enhances Nrf2 signaling to protect HepG2 cells from oxidative damage through Akt and ERK activation. Biomed Pharmacother.

[25] Du K, Ramachandran A, Jaeschke H (2016). Oxidative stress during acetaminophen hepatotoxicity: sources, pathophysiological role and therapeutic potential. Redox Biol.

[26] Jaeschke H, McGill MR, Williams CD, Ramachandran A (2011). Current issues with acetaminophen hepatotoxicity--a clinically relevant model to test the efficacy of natural products. Life Sci.

[27] Palliyaguru DL, Chartoumpekis DV, Wakabayashi N, Skoko JJ, Yagishita Y, Singh SV, Kensler TW (2016). Withaferin a induces Nrf2-dependent protection against liver injury: role of Keap1-independent mechanisms. Free Radic Biol Med.

[28] Kim MH, Kim EH, Jung HS, Yang D, Park EY, Jun HS (2017). EX4 stabilizes and activates Nrf2 via PKCdelta, contributing to the prevention of oxidative stress-induced pancreatic beta cell damage. Toxicol Appl Pharmacol.

[29] Zhou J, Ma X, Cui Y, Song Y, Yao L, Liu Y, Li S (2017). Methyleugenol protects against t-BHP-triggered oxidative injury by induction of Nrf2 dependent on AMPK/GSK3beta and ERK activation. J Pharmacol Sci.

[30] Jiang YM, Wang Y, Tan HS, Yu T, Fan XM, Chen P, Zeng H, Huang M, Bi HC (2016). Schisandrol B protects against acetaminophen-induced acute hepatotoxicity in mice via activation of the NRF2/ARE signaling pathway. Acta Pharmacol Sin.

[31] Song X, Yin S, Huo Y, Liang M, Fan L, Ye M (2015). Hu H: Glycycoumarin ameliorates alcohol-induced hepatotoxicity via activation of Nrf2 and autophagy. Free Radic Biol Med.

[32] Lv H, Liu Q, Wen Z, Feng H, Deng X, Ci X (2017). Xanthohumol ameliorates lipopolysaccharide (LPS)-induced acute lung injury via induction of AMPK/GSK3beta-Nrf2 signal axis. Redox Biol.

[33] Luo S, Li Z, Mao L, Chen S, Sun S. Sodium butyrate induces autophagy in colorectal cancer cells through LKB1/AMPK signaling. J Physiol Biochem. 2018:1–11. Epub ahead of print. 10.1007/s13105-018-0651-z. 10.1007/s13105-018-0651-z 30362049

[34] Vazirian M, Nabavi SM, Jafari S, Manayi A (2018). Natural activators of adenosine 5′-monophosphate (AMP)-activated protein kinase (AMPK) and their pharmacological activities. Food Chem Toxicol.

[35] Brodsky M, Hirsh S, Albeck M, Sredni B (2009). Resolution of inflammation-related apoptotic processes by the synthetic tellurium compound, AS101 following liver injury. J Hepatol.

[36] Huang H, Zhang X, Li J (2015). Protective effect of oroxylin a against lipopolysaccharide and/or D-galactosamine-induced acute liver injury in mice. J Surg Res.

[37] Schafer FQ, Buettner GR (2001). Redox environment of the cell as viewed through the redox state of the glutathione disulfide/glutathione couple. Free Radic Biol Med.

[38] Du K, Farhood A, Jaeschke H (2017). Mitochondria-targeted antioxidant Mito-tempo protects against acetaminophen hepatotoxicity. Arch Toxicol.

[39] Saito C, Lemasters JJ, Jaeschke H (2010). C-Jun N-terminal kinase modulates oxidant stress and peroxynitrite formation independent of inducible nitric oxide synthase in acetaminophen hepatotoxicity. Toxicol Appl Pharmacol.

[40] Sharifudin SA, Fakurazi S, Hidayat MT, Hairuszah I, Moklas MA, Arulselvan P (2013). Therapeutic potential of Moringa oleifera extracts against acetaminophen-induced hepatotoxicity in rats. Pharm Biol.

[41] Bataille AM, Manautou JE (2012). Nrf2: a potential target for new therapeutics in liver disease. Clin Pharmacol Ther.

[42] Gum SI, Cho MK (2013). Recent updates on acetaminophen hepatotoxicity: the role of nrf2 in hepatoprotection. Toxicol Res.

[43] Tsao SM, Yin MC (2015). Antioxidative and antiinflammatory activities of asiatic acid, glycyrrhizic acid, and oleanolic acid in human bronchial epithelial cells. J Agric Food Chem.

[44] Nicholls SJ (2008). The complex intersection of inflammation and oxidation: implications for atheroprotection. J Am Coll Cardiol.

